# Comprehensive High-Depth Proteomic Analysis of Plasma Extracellular Vesicle-Containing Preparations in CDKL5 Deficiency Disorder

**DOI:** 10.3390/biomedicines14050961

**Published:** 2026-04-22

**Authors:** Tadashi Shiohama, Satoru Takahashi, Ryo Takeguchi, Yuichi Akaba, Hironori Sato, Masaki Ishikawa, Yusuke Kawashima, Asuka Koshi, Chihiro Abe, Shin Nabatame, Keita Tsujimura, Hiromichi Hamada, Keiichiro Suzuki

**Affiliations:** 1 Department of Pediatrics, Graduate School of Medicine, Chiba University, Inohana 1-8-1, Chuo-ku, Chiba-shi 260-0856, Chiba, Japan; 2 Department of Pediatrics, International University of Health and Welfare Narita Hospital, Hatakeda 852, Narita 286-8520, Chiba, Japan; 3 Department of Pediatrics, Asahikawa Medical University, 2-1-1-1 Midorigaoka Higashi, Asahikawa City 078-8510, Hokkaido, Japan; 4 Department of Applied Genomics, Kazusa DNA Research Institute, Kisarazu 292-0818, Chiba, Japan; 5 Department of Pediatrics, Graduate School of Medicine, The University of Osaka, Suita 565-0871, Osaka, Japan; 6 Group of Brain Function and Development, Nagoya University Neuroscience Institute of the Graduate School of Science, Nagoya 464-8602, Aichi, Japan; 7 Research Unit for Developmental Disorders, Institute for Advanced Research, Nagoya University, Nagoya 464-8601, Aichi, Japan; 8 Institute for Advanced Co-Creation Studies, The University of Osaka, 1-3 Machikaneyama, Toyonaka 560-8531, Osaka, Japan; 9 Graduate School of Engineering Science, The University of Osaka, 1-3 Machikaneyama, Toyonaka 560-8531, Osaka, Japan; 10 Graduate School of Frontier Bioscience, The University of Osaka, 1-3 Yamadaoka, Suita 565-0871, Osaka, Japan

**Keywords:** CDKL5 deficiency disorder, extracellular vesicles-containing preparations, high-depth proteome analysis, AMPA, GABA

## Abstract

**Background/Objectives**: CDKL5 deficiency disorder (CDD) is a rare X-linked developmental and epileptic encephalopathy characterized by early onset refractory epilepsy and severe neurodevelopmental impairment with autistic features. Despite advances in genetic diagnosis, objective biomarkers reflecting disease mechanisms remain limited. Extracellular vesicles (EVs) circulating in the blood may contain disease-related proteins derived from the central nervous system. This study aimed to characterize the plasma EV proteome in CDD in a hypothesis-generating exploratory framework and identify the candidate molecular pathways associated with this disorder. **Methods**: Plasma samples from seven patients with genetically confirmed CDD and seven neurotypical developmental controls were analyzed. Extracellular vesicle-containing preparations (EVs-cp) were isolated via immunoprecipitation using antibodies against CD9, CD63, and CD81. Proteomic profiling was performed using data-independent mass spectrometry. Differentially expressed proteins were identified using Welch’s *t*-test with a false discovery rate correction. Functional enrichment, protein interaction network, and correlation analyses were performed using CDKL5 Clinical Severity Assessment (CCSA) scores. **Results**: In total, 5617 proteins were identified, of which 3510 were used for quantitative analysis. Compared to the controls, 2108 proteins were upregulated and 158 were downregulated in the CDD samples. Enrichment analysis revealed alterations in vesicle-mediated transport, cytoskeletal organization, and immune-related pathways. Several proteins were also correlated with clinical severity scores. **Conclusions**: Plasma EV proteomics revealed molecular alterations associated with CDD and provided a potential approach for biomarker discovery and mechanistic investigation.

## 1. Introduction

CDKL5 deficiency disorder (CDD) is an X-linked neurodevelopmental disorder caused by pathogenic variants of the *CDKL5* gene [1,2]. It is characterized primarily by early onset, treatment-resistant epilepsy that typically manifests within the first few weeks to two months of life and by severe developmental delay with autistic features. A wide range of systemic manifestations, including sleep disturbances, hypotonia, visual abnormalities, feeding and swallowing difficulties, gastroesophageal reflux, and scoliosis contribute to a highly heterogeneous clinical phenotype. Initially regarded as a variant of Rett syndrome, CDD is now recognized as a distinct developmental and epileptic encephalopathy.

Currently, there is no established disease-modifying therapy, and management relies mainly on symptomatic approaches such as polytherapy with anti-seizure medications, dietary interventions, and neuromodulation [3]. However, seizures are frequently intractable to treatments. Furthermore, persistent challenges include poor acquisition of motor and cognitive skills, and the need for long-term management of complications in the sleep, gastrointestinal, and orthopedic domains. Clinical heterogeneity, combined with temporal fluctuations in seizure frequency and developmental milestones and the lack of objective biomarkers of disease activity, complicate both the evaluation of therapeutic interventions and the design of clinical trials.

Recently, analysis of the blood proteome, particularly proteins contained within cell-derived exosomes (extracellular vesicles, EVs), has gained attention as a promising “liquid biopsy” method for capturing molecular information originating from the central nervous system (CNS) [4]. EVs are relatively stable in the circulation and enable the detection of brain-derived molecules that are otherwise difficult to access because of the blood–brain barrier, thereby allowing non-invasive detection of disease-specific molecular alterations. The discovery of EV-based biomarkers is advancing rapidly in the fields of neurodegenerative diseases and pediatric drug-resistant epilepsy. Blood-based proteomic profiling is a useful approach to comprehensively investigate the molecular pathways implicated in the pathogenesis of CDD, including synaptic dysfunction, mitochondrial impairment, and neuroinflammation/oxidative stress.

Cerebrospinal fluid (CSF) proteomic analyses have identified differentially expressed proteins that aid in distinguishing CDD from Rett syndrome [4]. At the plasma level, studies have reported altered inflammatory cytokine profiles and increased oxidative stress markers, such as 4-hydroxynonenal–protein adducts (4HNE-PAs), in CDKL5-related Rett syndrome [5]. Furthermore, functional protein analyses using iPSC-derived neurons and murine models of CDD revealed abnormalities in synaptic proteins [6]. However, comprehensive proteomic studies focusing on the exosomal fraction of plasma or serum remain limited, and objective and reliable biomarkers have not yet been established.

A major limitation of conventional plasma or serum proteomic analyses is the masking of low-abundance disease-specific proteins by highly abundant proteins such as albumin [7]. In contrast, the analysis of plasma EVs involves the enrichment of cell-derived proteins and reduces interference from non-specific, high-abundance proteins [8]. Moreover, because exosomal proteins are encapsulated within a lipid bilayer, they are protected from proteolytic degradation, providing enhanced stability, making them suitable for longitudinal monitoring of molecular changes [9]. These unique properties suggest that profiling CNS-derived EVs from the peripheral blood could facilitate the development of biomarkers that reflect disease activity and therapeutic responsiveness in patients with CDD.

Here, we performed a proteomic analysis of extracellular vesicle-containing preparations (EVs-cp) from plasma of patients with CDD to identify proteins that could serve as new biomarkers. Per Minimal Information for Studies of Extracellular Vesicles (MISEV) guidelines “https://www.isev.org/misev (accessed on 1 October 2024)”, EVs are defined as particles that are released from cells, delimited by a lipid bilayer, and cannot replicate on their own. EVs can cross the cerebrovascular barrier and are considered potential biomarkers of CNS diseases. Recent studies have successfully identified new biomarkers by analyzing EVs, including exosomes [10], and we postulated that a comparable investigation of EVs or EVs-cp in CDD may yield novel biomarkers of the disease. Using high-depth data-independent acquisition (DIA), we successfully identified over 2000 proteins.

## 2. Materials and Methods

### 2.1. Subjects and Collection of Plasma Samples

Seven patients who met the clinical and genetic diagnostic criteria for CDD were selected from the patient population of Asahikawa Medical University Hospital [11,12]. Neurotypical developmental controls (NCs) from the patient population at Asahikawa Medical University Hospital and Chiba University Hospital were also enrolled after obtaining written informed consent.

The CDKL5 Developmental Score (CDS) [13] was used to evaluate developmental status in patients with CDD. One point was assigned for each of the following milestones achieved: independent sitting, independent standing, independent walking, raking grasp, pincer grasp, babbling, and the ability to say a single word. The CDS ranges from 0 to 7, with higher scores reflecting the acquisition of a greater number of developmental milestones. An adapted form of the CDKL5 Clinical Severity Assessment (CCSA) [14] was applied to assess disease severity. The CCSA is structured as a categorical measure across six domains: seizure (0–21), motor (0–25), behavior, speech, and sleep (0–18), gastrointestinal (0–9), respiratory (0–8), and other comorbidities (0–2). The total CCSA score ranges from 0 to 83, where higher scores correspond to more severe clinical manifestations.

Blood samples were collected from seven patients with CDD, all of whom had clinically and genetically confirmed diagnoses, as well as from seven normal controls (NCs). Blood was drawn into complete RNA blood collection tubes (Streck, La Vista, NE, USA). Samples were first centrifuged at 1800× *g* for 15 min at room temperature. The resulting supernatant was subsequently centrifuged at 2800× *g* for an additional 15 min at room temperature and then promptly stored at −80 °C until further analysis.

### 2.2. Pipeline for Plasma EVs-cp Proteomics

#### 2.2.1. Purification of EVs-cp from Human Plasma

Human plasma EVs-cp were isolated by an immunoprecipitation-based approach using the ExoCap Streptavidin Kit (MBL Co., Ltd., Tokyo, Japan) and the Maelstrom 8 Autostage (Taiwan Advanced Nanotech Inc., Taoyuan, Taiwan). Initially, 250 µL of human plasma was centrifuged at 15,000× *g* for 30 min at 4 °C. The resulting supernatant (150 µL) was carefully transferred to new tubes. An equal volume (150 µL) of Treatment Buffer supplied with the kit was then added and gently mixed. For immunoprecipitation, biotinylated monoclonal antibodies against CD9, CD63, and CD81 (each at 1 mg/mL; MBL Co., Ltd.) were combined in equal proportions (1:1:1, *v*/*v*/*v*). Streptavidin magnetic beads (80 µL; Sera-Mag SpeedBeads Blocked Streptavidin Particles, 80 µg solids; Cytiva, Marlborough, MA, USA) were washed with 300 µL of Treatment Buffer. Subsequently, 2.5 µL of the antibody mixture was added to the washed beads in 200 µL of Treatment Buffer and incubated for 10 min. The beads were then washed twice with 200 µL of Treatment Buffer prior to addition to the prepared plasma samples. To facilitate binding of EVs-cp to the antibody-coated beads, the mixture was agitated at 1000 rpm for 2 h. After incubation, the beads were washed three times with 500 µL of Washing/Dilution Buffer (provided with the ExoCap Streptavidin Kit. Finally, EVs-cp bound to the beads were eluted using 100 µL of buffer containing 100 mM Tris-HCl (pH 8.0), 20 mM NaCl, and 4% SDS.

#### 2.2.2. Sample Preparation Proteome Analysis

Protein extracts were first reduced with 20 mM tris (2-carboxyethyl) phosphine at 80 °C for 10 min, followed by alkylation with 30 mM iodoacetamide at room temperature for 30 min in the dark. Protein purification and enzymatic digestion were carried out using the sample preparation (SP3) method [15,16,17]. Tryptic digestion was performed overnight at 37 °C using Trypsin/Lys-C Mix (Promega, Madison, WI, USA) at a concentration of 500 ng/µL. The resulting peptide mixtures were purified with GL-Tip SDB (GL Sciences, Tokyo, Japan) according to the manufacturer’s instructions. Finally, peptides were resuspended in 2% acetonitrile containing 0.1% trifluoroacetic acid.

### 2.3. LC-MS/MS

The digested peptides were directly introduced onto a C18 nano-capillary column (IonOpticks, VIC, Australia; 75 µm × 25 cm) maintained at 60 °C. Peptide separation was achieved over a 100-min gradient using mobile phase A (0.1% formic acid in water) and mobile phase B (0.1% formic acid in 80% acetonitrile). The gradient profile was set as follows: 7% B at 0 min, increasing to 37% B at 86 min, then to 70% B at 93 min, and maintained at 70% B until 100 min. The flow rate was fixed at 150 nL/min using an UltiMate 3000 RSLCnano LC system (Thermo Fisher Scientific, Waltham, MA, USA).

Eluted peptides were analyzed on a quadrupole Orbitrap Exploris 480 hybrid mass spectrometer (Thermo Fisher Scientific) operated in DIA mode with standard window settings. MS1 spectra were acquired over an *m*/*z* range of 495–745 at a resolution of 60,000. The automatic gain control (AGC) target for MS1 was set to 3 × 10^6^, with the maximum injection time configured to “auto.”

MS2 spectra were collected across an *m*/*z* range of 200–1800 at a resolution of 45,000, with an AGC target of 3 × 10^6^ and maximum injection time set to “auto.” A normalized collision energy of 26% was applied. The MS2 isolation window was set to 4 Th, and for the *m*/*z* range of 500–740, an optimized window scheme was implemented using Scaffold DIA version 3.3.1 (Proteome Software, Inc., Portland, OR, USA).

### 2.4. Data Processing

Raw data were analyzed using DIA-NN [18] (version 1.8.1; “https://github.com/vdemichev/DiaNN (1 March 2023)” against an in silico-generated spectral library. The spectral library was constructed within DIA-NN based on the Human UniProtKB/Swiss-Prot database (UniProt ID: UP000005640; 20,588 reviewed canonical entries; released 11 November 2021). The DIA-NN search was performed with the following parameters: trypsin as the protease, allowing up to one missed cleavage; peptide length range of 7–45 amino acids; pre-cursor charge states of 2–4; precursor *m*/*z* range of 490–750; Fragment ion *m*/*z* range of 200–1800; MS1 and MS2 mass accuracies set to 10 ppm; and carbamidomethylation of cysteine specified as a fixed modification. The options “heuristic protein interferences,” “no shared spectra,” “Unrelated runs,” and “use isotopologues” were enabled. Protein identification was filtered at a false discovery rate (FDR) of <1% at both the precursor and protein levels. Protein intensities were calculated using the Robust LC (high precision) mode, and protein intensities across samples were normalized using the RT-dependent mode. Protein intensities were log2-transformed, and data were filtered to retain proteins with at least 70% valid values in at least one group. Missing values were imputed using random numbers drawn from a normal distribution (width = 0.3, down-shift = 2.8) in Perseus (version 2.0.11) [19]. Differentially expressed proteins were defined as those showing more than a two-fold change with *p* < 0.05 (Welch’s *t*-test) between the two groups, and volcano plot and heatmap were generated based on these criteria. Details of the DIA window scheme and the overall data processing workflow are provided in Table S1 and Figure S1, respectively.

### 2.5. Use of Artificial Intelligence

Chat GPT-5.2 (OpenAI, Abilene, TX, USA) was used for language editing and summarizing literature. The tool was accessed from November 2025 to March 2026.

## 3. Results

All patients with CDD exhibited de novo onset and pathogenic heterozygous variants (CDKL5-01, CDKL5-02, CDKL5-03, CDKL5-04, CDKL5-05, CDKL5-06, and CDKL5-07). The CDS and Autism Spectrum Screening Checklist (total and individual domains) scores of patients were summarized in Table 1. The NCs participants (Ctrl-01 Female, 16y9m; Ctrl-02 Female, 13y11m; Ctrl-03 Female, 13y11m; Ctrl-04 Female, 10y11m; Ctrl-05 Female, 6y11m; Ctrl-06 Male, 6y2m; Ctrl-07 Male, 13y2m). The CDS and CCSA scores for each NCs participants were zero. The ages of the CDD and NCs participants were also matched using Welch’s *t*-test (*t* = 0.57, *p* = 0.586).

A comprehensive DIA-based proteomic analysis of plasma samples obtained from 14 individuals (seven patients with CDD and seven NCs) identified a total of 5617 proteins (Table 1). Data processing was conducted using Perseus (version 2.0.11), and 3510 proteins were retained for further analysis based on the criterion that at least two unique peptides were detected in more than 70% of samples in both the CDD and NC groups.

**Figure 1:**
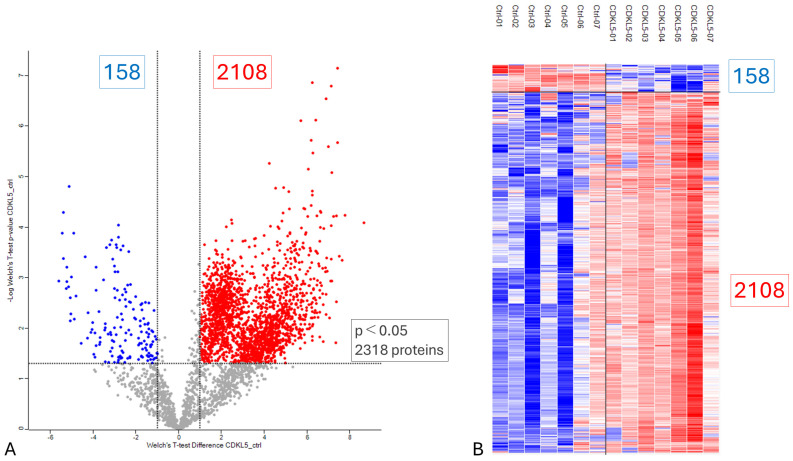
(**A**) Volcano plot illustrating the upregulated and downregulated proteins differentially expressed between patients with CDD and the NC group. Each data point represents an individual protein, characterized by both the magnitude of change (log_2_ fold change, *x*-axis) and statistical significance (−log_10_ *p*-value, *y*-axis). The horizontal threshold line corresponds to *p* = 0.05, while the vertical threshold lines indicate fold changes of 2 and 0.5. Proteins with fold changes ≥2 are highlighted as red squares, whereas those with fold changes ≤0.5 are shown as blue squares. (**B**) Heatmap with hierarchical clustering (dendrogram) depicting proteins that are significantly different between the CDD and NC groups. Log-transformed protein expression values were normalized using z-score scaling and visualized with corresponding color coding.

An F-test indicated unequal variances between the two groups; therefore, group comparisons were performed using Welch’s *t*-test. Missing values were imputed using a width of 0.3 and a downshift of 2.8. Differential expression analysis was carried out with Welch’s *t*-test followed by Benjamini–Hochberg false discovery rate (FDR) correction (q = 0.05). Proteins exhibiting a fold change ≥2 were defined as upregulated (n = 2108; red), whereas those with a fold change ≤0.5 were considered downregulated (n = 158; blue) (Figure 1A and Table S2). Proteins showing statistically significant differences between the two groups are visualized in the heatmap and corresponding dendrogram presented in Figure 1B.

**Figure 2:**
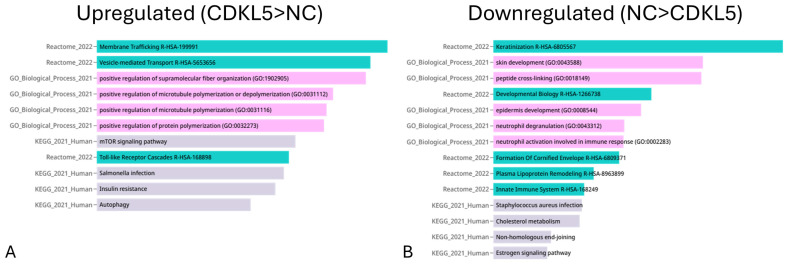
Enrichment analysis by Enrichr-KG, utilizing databases of the GO Biological Process, KEGG pathway, and Reactome pathway with the upregulated proteins in the top 100 by fold change (**A**) and the 158 downregulated proteins (**B**) between CDKL5 deficiency and neurotypical developmental control groups.

The upregulated proteins in the top 100 by fold change (Figure 2A) and the 158 downregulated proteins (Figure 2B) were analyzed using Enrichr-KG (https://maayanlab.cloud/enrichr-kg) as follows: FDR cutoff of q = 0.05, pathway size of 2–5000, and the options “remove redundancy” and “abbreviate pathways” enabled. The upregulated pathways included membrane trafficking, vesicle-mediated transporters, supramolecular fibers, microtubule organization, the mTOR pathway, the toll-like receptor cascade, insulin resistance, and autophagy. The downregulated pathways included skin development (keratinization, epidermis, and cornified envelope), developmental biology, and neutrophil function.

**Figure 3:**
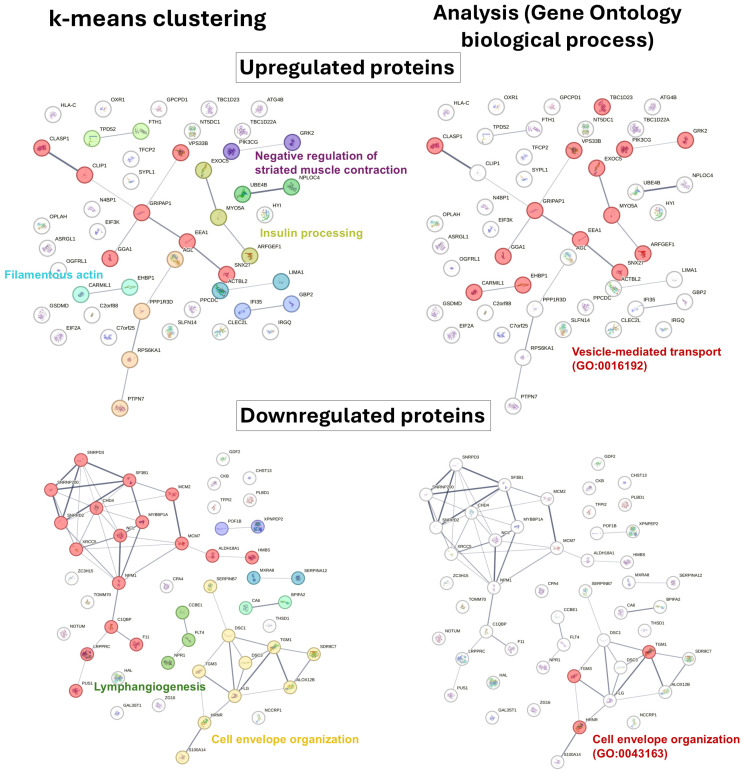
Interaction network mapping of top 50 upregulated (upper panels) and downregulated (lower panels) proteins in fold change analyzed using STRING (version 12.0). The line color indicates the type of interaction evidence as specified in the key. Functional clusters were identified using STRING clustering analysis, and major network modules are annotated accordingly. Interaction confidence was set to a medium confidence score (0.4).

In addition, protein interactions were analyzed among the top 50 proteins in terms of fold change in upregulated (Figure 3, Table S3) and downregulated proteins (Figure 3, Table S3) using STRING (https://string-db.org/) [20]. Differentially expressed proteins identified in CDKL5 deficiency disorder (CDD) were analyzed using STRING (version 12.0) to construct protein–protein interaction networks. Interaction confidence was set to a medium confidence score (0.4). The k-means clustering (Figure 3, left panels) was applied to the interaction networks to identify functional clusters among upregulated (Figure 3, upper panels) and downregulated (Figure 3, lower panels) proteins. Representative biological themes associated with each cluster are indicated. Gene Ontology (GO) biological process enrichment analysis (Figure 3, right panels) was performed using STRING. The most significantly enriched pathways are highlighted, including vesicle-mediated transport (GO:0016192) in upregulated proteins and cell envelope organization (GO:0043163) in downregulated proteins.

**Figure 4:**
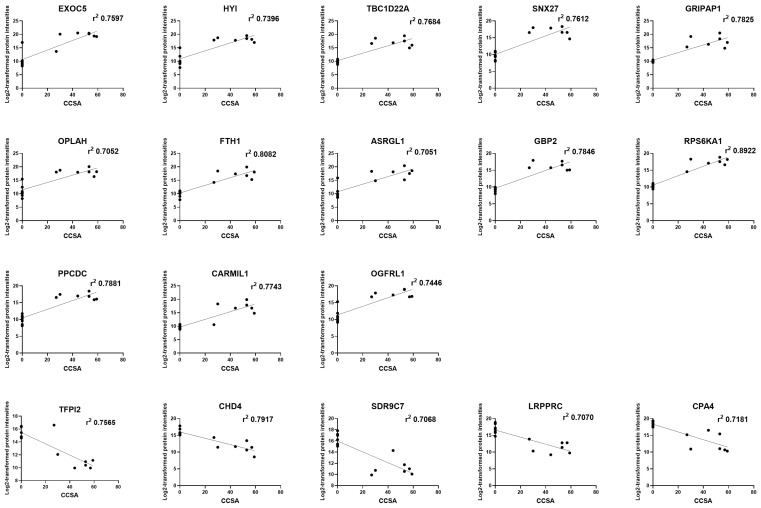
Correlation between protein expression levels and clinical severity in CDKL5 deficiency disorder. Scatter plots showing the relationship between protein expression levels and CDKL5 Clinical Severity Assessment (CCSA) scores. Each point represents an individual sample. The *y*-axis indicates log2-transformed protein intensities derived from DIA-NN output, reflecting MS2-based quantification at the protein level. The *x*-axis represents CCSA scores. Linear regression analysis was performed to evaluate the association between protein expression levels and clinical severity, and the coefficient of determination (r^2^) is indicated for each protein. Proteins with strong correlations (r^2^ > 0.7) are shown.

Next, we focused on the correlation between clinical severity and protein expression levels. Protein expression levels were quantified as log2-transformed protein intensities derived from DIA-NN output. We evaluated the relationship between CCSA and the log2-transformed protein intensities of the top 50 upregulated and downregulated proteins using simple linear regression (Tables S3 and S4). A correlation coefficient (r) > 0.7 (r^2^ > 0.5) was defined as indicative of a highly correlated relationship [21,22]. Of the 100 proteins examined, 57 had r^2^ values of at least 0.5, 35 had r^2^ values of at least 0.6, and 18 had r^2^ values of at least 0.7, without applying multiple testing correction (Table S4). Eighteen proteins with r^2^ values of at least 0.7, including 13 (EXOC5, HYI, TBC1D22A, SNX27, GRIPAP1, OPLAH, FTH1, ASRGL1, GBP2, RPS6KA1, PPCDC, CARMIL1, and OGFRL1) from the upregulated group and five (TFPI2, CHD4, SDR9C7, LRPPRC, and CPA4) from the downregulated group, exhibited a strong correlation with CCSA (Figure 4).

## 4. Discussion

Through DIA proteomic analysis of plasma-isolated EVs-cp, we identified 2108 upregulated and 158 downregulated proteins in participants with CDD compared to those in NC. The relatively large number of differentially expressed proteins may reflect not only biological differences but also global shifts in proteomic profiles or technical factors related to normalization and preprocessing. Therefore, these findings should be interpreted cautiously, particularly in the context of the limited sample size.

Human plasma EVs-cp were isolated by an immunoprecipitation-based approach. It should be noted that the immunoprecipitation-based approach using tetraspanin markers enriches vesicle-associated components but does not ensure complete specificity or purity. At the same time, the reliability of our purification pipeline for EVs-cp from human plasma was demonstrated through comparison with proteomic signatures reported by other investigators [23] and the Vesiclepedia database [24] in our previous report [15].

Enrichment analysis using the top 100 upregulated and downregulated proteins with the largest fold change identified fluctuating concentrations of proteins functionally involved in synaptic trafficking, mitochondrial function, and neuroinflammation, instead of structural proteins. These findings may provide insights into the molecular pathways potentially associated with CDD, extending the findings by placing the observed protein alterations within the established neuropathological frameworks of developmental epileptic encephalopathies. At the same time, it is important to note that the EVs-cp analyzed in this study likely represent a mixture of vesicles derived from multiple tissues. As no neuron-specific enrichment or validation was performed, the central nervous system origin of the detected proteins cannot be confirmed. Therefore, interpretations related to synaptic and neuronal pathways should be considered biologically plausible but not definitive.

Among upregulated proteins, the interaction network revealed several interconnected clusters associated with vesicle-mediated transport, cytoskeletal organization, and metabolic processes. Gene Ontology (GO) enrichment analysis identified vesicle-mediated transport (GO:0016192) as a major enriched biological process, supporting alterations in intracellular trafficking pathways. k-means clustering further highlighted functional modules, including those related to insulin processing, filamentous actin organization, and regulation of muscle contraction. In contrast, downregulated proteins formed distinct clusters enriched for processes related to cell envelope organization (GO:0043163) and lymphangiogenesis, suggesting alterations in structural and developmental pathways. Overall, these network-based analyses indicate that dysregulation of vesicle trafficking and cytoskeletal dynamics may represent key features of the proteomic alterations observed in CDD.

### 4.1. Synaptic Trafficking and Network Dysfunction in CDD

In this study, we identified coordinated upregulation of proteins involved in receptor trafficking and endosomal recycling, including SNX27, GRIPAP1, ARFGEF1, EXOC5, TBC1D22A/TBC1D23, CLIP1, and CLASP1. These pathways are central to the regulation of excitatory and inhibitory synaptic transmission, and are highly relevant to the pathophysiology of CDD. CDKL5 is a serine/threonine kinase required for synapse maturation and maintenance of excitatory–inhibitory balance, and its loss results in impaired dendritic spine stability and network hyperexcitability [25,26].

SNX27 and GRIPAP1 are critical regulators of AMPA recycling and activity-dependent synaptic plasticity. Disruption of these proteins impairs long-term potentiation and cognitive function, providing a mechanistic link between altered proteomic profiles and synaptic dysfunction in CDD [27,28,29]. The concurrent upregulation of ARFGEF1, a gene implicated in developmental epileptic encephalopathies, further supports a disturbance in inhibitory synapse regulation and GABA_receptor trafficking [30].

### 4.2. Cytoskeletal and Vesicular Transport Mechanisms

The increased abundance of EXOC5, CLIP1, and CLASP1 suggests broader disruption of cytoskeletal dynamics and vesicular transport. These proteins are essential for neuronal polarization, axonal growth, and synaptic vesicle targeting, and are particularly vulnerable during early brain development [31]. The genetic associations of CLIP1 and CLASP1 with severe neurodevelopmental disorders and epilepsy underscore the clinical relevance of this axis.

### 4.3. Mitochondrial Dysfunction as a Disease Modifier

In contrast to the upregulated trafficking-related proteins, C1QBP and LRPPRC were significantly downregulated. Both of these proteins are indispensable for mitochondrial RNA processing, translation, and oxidative phosphorylation. Pathogenic variants of LRPPRC cause Leigh syndrome and are associated with neurodevelopmental regression and epilepsy [32,33]. These findings suggested that impaired mitochondrial energy metabolism may act as a disease modifier, exacerbating neuronal vulnerability and seizure susceptibility in patients with CDD.

### 4.4. Neuroinflammatory Signaling

We also observed increased levels of the interferon-related proteins IFI35 and GBP2, indicating activation of innate immune and inflammatory pathways. Neuroinflammation has emerged as an important contributor to epileptogenesis and synaptic remodeling in multiple epilepsy syndromes, including developmental epileptic encephalopathies [34]. Previous studies have reported microglial activation in CDD, supporting the relevance of inflammatory signaling in disease progression [35].

CDD shares clinical similarities with Rett syndrome caused by MECP2 gene mutations, despite having unrelated causative genes [2,5]. Interestingly, our DIA proteomic study of Rett syndrome revealed the upregulation of inflammation-associated pathways, such as extracellular matrix–receptor interaction and focal adhesion, instead of decreased structural proteins [15]. The commonalities observed in the EVs-cp proteomic results for Rett and CDD syndromes may be related to the commonalities in their clinical presentations.

### 4.5. Limitations

This study had several limitations. First, a proteomic analysis was performed on circulating EVs, which may reflect a mixture of central and peripheral sources. Second, the cross-sectional design limits inferences regarding the temporal relationships between proteomic changes and clinical severity. Third, functional validation in patient-derived neuronal models is required to establish causal links between altered protein expression and synaptic or network dysfunction. In this study, orthogonal validation of EVs-cp, such as transmission electron microscopy, nanoparticle tracking analysis, or immunoblotting for canonical EV markers, was not performed due to limited sample availability. Extracellular vesicle-containing preparations typically yield limited protein quantities, particularly in rare disease cohorts, which constrains downstream validation analyses [9,16]. Validation of key candidate proteins (e.g., SNX27, LRPPRC) will be an important focus of future studies.

In addition, genetic and clinical heterogeneity was not fully accounted for in this study. Variability in CDKL5 variants, X-chromosome inactivation status in female patients, and potential somatic mosaicism were not analyzed. Furthermore, clinical factors such as seizure burden, treatment regimens, disease duration, and comorbidities were not controlled for. The small sample size inherent to rare disease research limits statistical power and generalizability. Therefore, the present findings should be considered hypothesis-generating.

## 5. Conclusions

We performed a DIA proteomic analysis of EV-cp samples extracted from the plasma of patients with CDD. A neurologically oriented interpretation of proteomic alterations in CDD suggested that alterations in synaptic transport may represent a potential pathomechanistic feature, supporting a multiaxial disease model involving the combined involvement of mitochondrial dysfunction and neuroinflammatory signaling. These findings provide a biologically coherent framework for future biomarker development and the identification of therapeutic targets.

## Figures and Tables

**Table 1:** Clinical background of patients with CDKL5 Deficiency Disorder.

	CDKL5-01	CDKL5-02	CDKL5-03	CDKL5-04	CDKL5-05	CDKL5-06	CDKL5-07
Age	23y4m	26y	8y	11y4m	8y	5y3m	12y6m
Sex	Female	Female	Male	Female	Female	Female	Male
Pathogenic variant in *CDKL5* (nucleotide, protein)	c.2697_2701dupAGCCC, p.(Leu901Glnfs*28)	c.2112C>A, p.(Tyr704*)	c.125A>G, p.(Lys42Arg)	c.530A>G, p.(Tyr177Cys)	c.532C>T, p.(Arg178Trp)	c.2026del, p.(His676Ilefs*108)	c.533G>A, p.(Arg178Gln)
CDS (total score 7)	5	0	0	0	3	0	0
CCSA (Overall total 83)	27	59	53	44	30	53	57
Epilepsy (0–21)	11	13	13	14	13	15	15
Motor (0–25)	2	22	20	20	8	22	20
Behavior, speech and sleep (0–18)	6	15	11	7	6	9	11
Gastrointestinal and feeding (0–9)	4	4	4	1	2	3	6
Respiratory (0–8)	2	3	3	0	0	3	3
Other comorbidities (0–2)	2	2	2	2	1	1	2

Nucleotides and amino acid residues were numbered according to the GenBank reference sequences (NM_003159.2 and NP_003150.1). The CDS ranges from 0 to 7, with higher scores reflecting greater acquisition of developmental milestones. The CCSA score, which consists of six domains, ranges from 0 to 83; a higher score indicates more severe clinical symptoms. Abbreviations: CCSA, an adapted version of the CDKL5 Clinical Severity Assessment; CDS, CDKL5 Developmental Score.

## Data Availability

The original contributions presented in the study are included in the article/Supplementary Material. Further inquiries can be directed at the corresponding author.

## Supplementary Materials

The following supporting information can be downloaded at https://www.mdpi.com/article/10.3390/biomedicines14050961/s1, Table S1. Isolation windows optimized by Scaffold DIA, Table S2. List of proteins identified with statistically significant differences between CDD and control groups, Table S3. The list of top 50 proteins in fold change, Table S4. Correlation analysis between protein expression levels and CCSA scores, Figure S1. Overview of data processing workflow including DIA-NN and Perseus analysis.

## Abbreviations

CDD, CDKL5 deficiency disorder; EVs-cp, extracellular vesicle-containing preparations; CDS, CDKL5 Developmental Score; CCSA, CDKL5 Clinical Severity Assessment.
