# Supplementary material for: Comprehensive High-Depth Proteomic Analysis of Plasma Extracellular Vesicle-Containing Preparations in CDKL5 Deficiency Disorder
Source: Biomedicines. 2026 Apr 22;14(5):961. doi: 10.3390/biomedicines14050961 (PMC13203274; doi:10.3390/biomedicines14050961)
Supplement: Supplementary file 1 [file biomedicines-14-00961-s001.zip › Supplementary data_proof_20260420/Table S3.docx]

| **Table S3.** The list of top 50 proteins in fold change | |
| --- | --- |
|  | **Gene symbol (log2 fold change)** |
| Upregulated | EXOC5 (8.67), HYI (7.79), CLASP1 (7.65), ARFGEF1 (7.5), TBC1D22A (7.44), SNX27 (7.44), IFI35 (7.39), GRIPAP1 (7.37), IGHV1-2 (7.36), OPLAH (7.24), CLIP1 (7.18), FTH1 (7.18), ASRGL1 (7.14), TPD52 (7.14), GBP2 (7.13), TBC1D23 (7.12), PTPN7 (7.06), RPS6KA1 (7.01), EEA1 (6.95), PIK3CG (6.92), PPCDC (6.9), NPLOC4 (6.9), HLA-C (6.9), NT5DC1 (6.87), CARMIL1 (6.86), ATG4B (6.79), GRK2 (6.78), N4BP1 (6.75), C7orf25 (6.74), LIMA1 (6.74), GSDMD (6.68), UBE4B (6.67), EHBP1 (6.63), OGFRL1 (6.63), TFCP2 (6.62), GPCPD1 (6.61), C2orf88 (6.61), AGL (6.61), GGA1 (6.59), ACTBL2 (6.59), IRGQ (6.57), CLEC2L (6.56), MYO5A (6.53), OXR1 (6.5), VPS33B (6.5), SYPL1 (6.48), SLFN14 (6.48), EIF2A (6.47), PPP1R3D (6.45), EIF3K (6.43) |
| Downregulated | CPA4 (–5.61), ZC3H15 (–5.43), LRPPRC (–5.4), BPIFA2 (–5.39), POF1B (–5.27), S100A14 (–5.24), MYBBP1A (–5.23), MCM7 (–5.16), SDR9C7 (–5.11), NPM1 (–5.07), SNRNP200 (–5.06), SERPINA12 (–5.03), HAL (–5.03), SNRPD3 (–4.9), XPNPEP2 (–4.88), SNRPD2 (–4.79), TGM1 (–4.55), CHD4 (–4.38), NCCRP1 (–4.25), NPR1 (–4.24), SF3B1 (–4.09), ZG16 (–4.06), GAL3ST1 (–4.02), ALDH18A1 (–3.96), APLP1 (–3.92), XRCC5 (–3.91), TOMM70 (–3.87), STXBP6 (–3.87), HRNR (–3.86), TFPI2 (–3.73), CCBE1 (–3.71), MCM2 (–3.68), CHST13 (–3.68), ALOX12B (–3.51), CA6 (–3.45), PLBD1 (–3.44), HTRA1 (–3.42), GDF2 (–3.42), DSC3 (–3.37), F11 (–3.28), LALBA (–3.27), DSC1 (–3.21), FLT4 (–3.2), NOTUM (–3.18), PUS1 (–3.18), NCL (–3.13), SERPINB7 (–3.11), CLEC10A (–3.11), THSD1 (–3.1), C1QBP (–3.1) |
