# Supplementary figures and images for: Comprehensive High-Depth Proteomic Analysis of Plasma Extracellular Vesicle-Containing Preparations in CDKL5 Deficiency Disorder
Source: Biomedicines. 2026 Apr 22;14(5):961. doi: 10.3390/biomedicines14050961 (PMC13203274; doi:10.3390/biomedicines14050961)

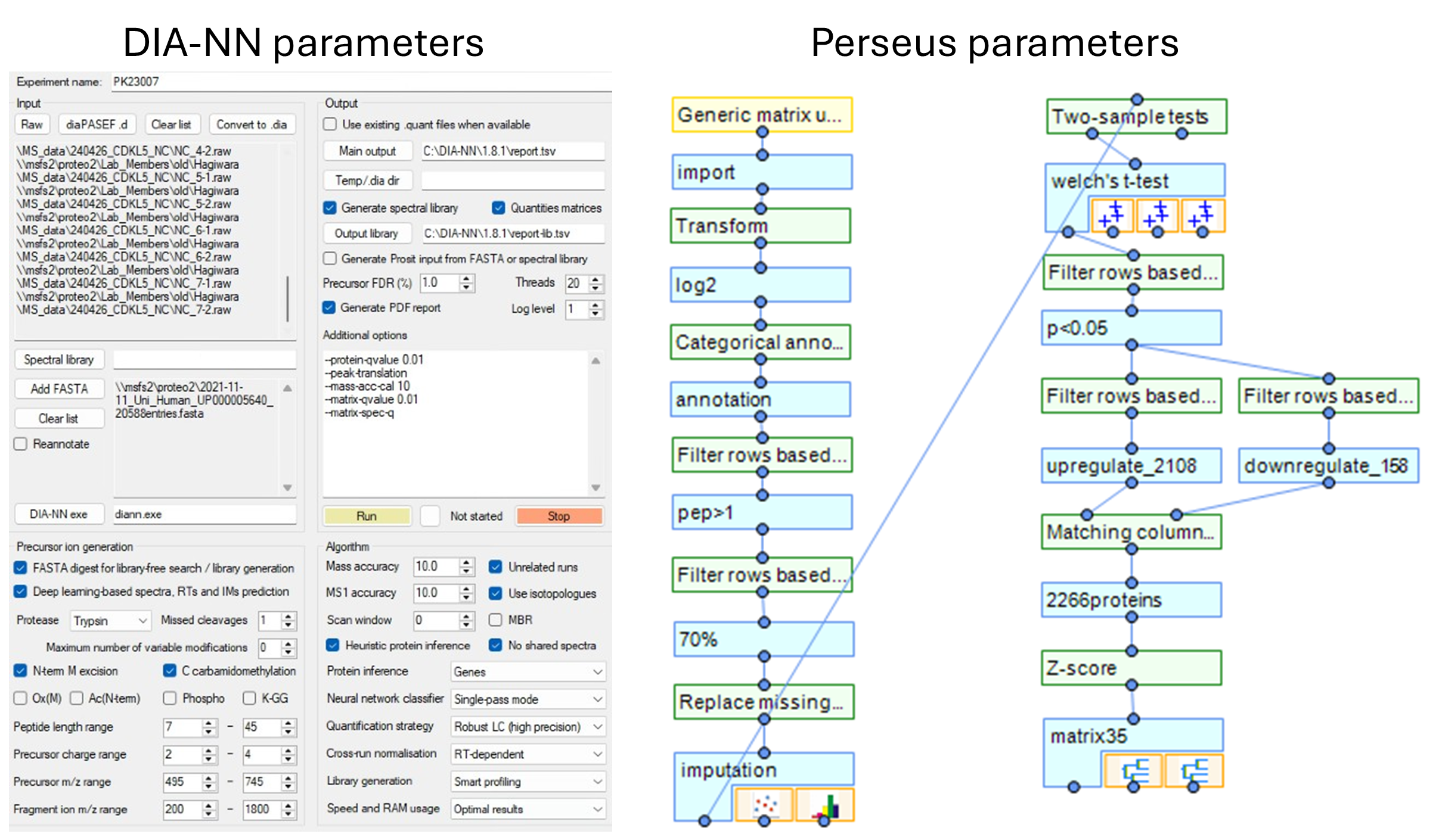

Supplement: Supplementary file 1 [file biomedicines-14-00961-s001.zip › Supplementary data_proof_20260420/Figure S1.tif]
